# From Accra to America: Putting development first in global health

**DOI:** 10.1371/journal.pgph.0007343

**Published:** 2026-09-18

**Authors:** Jirair Ratevosian, William Nii Ayitey Menson

**Affiliations:** 1 Duke Global Health Institute, Duke University, Durham, North Carolina, United States of; 2 ONE Campaign, Abuja, Nigeria; PLOS: Public Library of Science, UNITED STATES OF AMERICA

When leaders gathered in Ghana for the 2025 Africa Health Sovereignty Summit, their message was clear: African governments want greater control over health financing, regional institutions, manufacturing, data, and global policy. The Accra Reset that ultimately emerged from that process was a demand to rethink who holds authority in global health and how that authority is exercised in countries [1].

One month later, the United States released its America First Global Health Strategy, emphasizing bilateral agreements, country co-investment, measurable results, and eventual self-reliance [2]. Early analysis of the strategy warned that a retreat from multilateralism, an overly geopolitical framing, and rushed transitions could threaten progress unless paired with credible country transition plans and sustained regional public goods.[3,4]

One year on, the two agendas offer a useful test. They share an interest in promoting national ownership, but not yet the same vision of sovereignty. The question is whether Washington’s new approach will help countries exercise greater control over their health systems, or simply transfer financial and operational risk to governments, facilities, workers, and patients before they have the resources and capacity to manage it.

The United States should welcome greater African agency while taking a candid view of PEPFAR’s legacy. Although the program is credited with saving more than 26 million lives [5], its achievements coexisted with structural limitations in the broader aid architecture. Built for an emergency, the legacy model relied on centralized decision-making to deliver results quickly, but remained top-down and administratively burdensome long after the emergency phase. Meanwhile, domestic financing commitments made by many African governments frequently went unmet. The task now is to preserve what worked while building a development-first partnership that puts countries in the lead and strengthens the national and regional systems needed to sustain progress.

## Bilateralism is useful, but insufficient

The America First strategy responds to genuine weaknesses in the previous model. It prioritizes country commitments, frontline commodities and health workers, measurable results, and self-reliance. These are reasonable objectives. Bilateral agreements can clarify responsibilities, align external support with national plans, and make governments harder to ignore.

Although branded as bilateral assistance, many agreements combine government-to-government financing with existing implementation mechanisms and support from local partners. Even so, they cannot replace the regional and multilateral cooperation needed to build and sustain laboratories, pooled procurement, manufacturing markets, surveillance networks, and regulatory systems that serve all countries.

At the country level, as Kaberuka and colleagues have argued, “domestic financing must be the foundation, not a footnote.” [6] That principle is sound, but financing alone does not create sovereignty. Sovereignty becomes meaningful when budgets are released, services are protected, procurement systems function, health workers are paid, and patients are not asked to finance the transition at the hospital gate.

If Washington is serious about promoting country ownership, it should recognize Africa CDC, the African Union, regional economic communities, the African Medicines Agency, and regional procurement platforms as central to shaping Africa’s health agenda. This is increasingly important as China, Saudi Arabia, and the UAE expand their economic and diplomatic engagement across the continent. Strong regional institutions with sustained financing and better coordination can strengthen outbreak preparedness and help countries secure quality-assured medical products [7,8].

The next phase of U.S. global health policy should work at two connected levels. At the national level, governments and civil society should set priorities and define domestic responsibilities and external support. At the regional level, funding should support functions that countries cannot efficiently provide alone, including procurement, regulation, manufacturing, surveillance, and emergency response.

## Sovereignty requires shared accountability

Sovereignty should be judged by results, not declarations [9]. The test is whether governments release committed budgets, medicines remain available, health workers are paid, outbreaks are detected, and patients are protected from rising costs.

National governments should have greater control over program design and delivery, while external partners should stop creating parallel systems and duplicative reporting. But greater flexibility must come with clear accountability for results, stronger public oversight, and firm protections for human rights.

That accountability should take the form of a focused set of public, measurable commitments for governments and external partners. These should cover financing, continuity of essential services, health-worker integration, commodity delivery, and priority health outcomes. These measures should also track what households pay to ensure the transition does not shift costs onto patients and families. Evidence from Ghana and Nigeria shows that costs previously covered by donors can migrate to patients when domestic systems are not ready to absorb them [10,11].

This risk becomes more important as countries integrate HIV, tuberculosis, malaria, immunization, primary health care, laboratories, supply chains, and community platforms. Integration may reduce duplication and expand access to services. However, without adequate financing, it can weaken the specialized services needed to reach specific populations and mask deterioration in programme performance [12].

People living with HIV, key populations, pregnant women, adolescents, tuberculosis patients, migrants, and people who depend on community-led services are often the first to disappear inside national averages. Civil society and community-led monitoring should therefore have an institutional role in measuring progress and turning local data into accountability [13].

## What a development-first strategy requires

Development-first means protecting health services today while building the national and regional capacity to sustain them over time. The U.S. Congress should exercise greater vigilance and stewardship as these aid transitions proceed. It should insist on predictable funding, transparent timelines, disease-specific outcome floors, community integration, and sustained investment in regional public goods that no bilateral agreement can finance alone.

Grants will continue to matter, especially in poorer countries and during health emergencies, including in conflict settings. Domestic financing cannot be discussed as though all governments are operating on a level field. Many African countries are trying to protect health spending while contending with high debt service, narrow tax bases, expensive borrowing, and limited fiscal space [14].

PEPFAR, USAID-supported health systems, the Global Fund, Gavi, and other partnerships have generated diplomatic credibility, scientific collaboration, outbreak intelligence, procurement influence, and greater regional stability. Multilateral institutions have not necessarily diluted American influence. At their best, they have extended it.

A development-first strategy must address the wider economic conditions that determine whether countries can sustain their health systems. African manufacturers need long-term purchase commitments to remain viable. Regional surveillance systems need clear rules for secure cross-border data sharing. Governments need affordable financing to purchase commodities on time [8]. The Development Finance Corporation can mobilize investment in manufacturing, diagnostics, infrastructure, and supply chains. A renewed and modernized AGOA can help African producers build reliable markets, while U.S. research partnerships and influence at the IMF, World Bank, and G20 can advance innovation and debt solutions that protect health spending.

The United States will not remain competitive by offering less while demanding more. It is strongest when it helps countries solve problems they have identified, mobilizes public and private capital, and supports institutions that last through changes of government in Washington and partner countries.

One year into the America First strategy, the number of agreements signed should not be the measure of progress. The real test is whether the new approach strengthens national health systems, makes regions safer, and protects people from losing care or facing higher costs during the transition. That is what a development-first strategy should deliver.
